# CD8 signaling in microglia/macrophage M1 polarization in a rat model of cerebral ischemia

**DOI:** 10.1371/journal.pone.0186937

**Published:** 2018-01-17

**Authors:** Jan Boddaert, Kenny Bielen, Bart ’s Jongers, Ekta Manocha, Laetitia Yperzeele, Patrick Cras, Daniel Pirici, Samir Kumar-Singh

**Affiliations:** 1 Molecular Pathology Group, Cell Biology and Histology, Faculty of Medicine and Health Sciences, Wilrijk, Belgium; 2 Department of Neurology, Universitair Ziekenhuis Antwerpen, Edegem, Belgium; 3 Translational Neuroscience – Faculty of Medicine and Health Sciences, Wilrijk, Belgium; 4 Department of Research Methodology, University of Medicine and Pharmacy of Craiova, Craiova, Romania; Charite Universitatsmedizin Berlin, GERMANY

## Abstract

Classical or M1 activity of microglia/macrophages has been described in several neurodegenerative and brain inflammatory conditions and has also been linked to expansion of ischemic injury in post-stroke brain. While different pathways of M1 polarization have been suggested to occur in the post-stroke brain, the precise underlying mechanisms remain undefined. Using a transient middle cerebral artery occlusion (MCAO) rat model, we showed a progressive M2 to M1 polarization in the perilesional brain region with M1 cells becoming one of the dominant subsets by day 4 post-stroke. Comparing key receptors involved in M1 polarization (CD8, IFNγR, Clec4, FcγR, TLR3 and TLR4) and their signal transducers (Syk, Stat1, Irf3, and Traf6) at the day 4 time point, we showed a strong upregulation of CD8 along with SYK transducer in dissected perilesional brain tissue. We further showed that CD8 expression in the post-stroke brain was associated with activated (CD68+) macrophages and that progressive accumulation of CD8+CD68+ cells in the post-stroke brain coincided with increased iNOS (M1 marker) and reduced Arg1 (M2 marker) expression on these cells. *In vitro* ligand-based stimulation of the CD8 receptor caused increased iNOS expression and an enhanced capacity to phagocytose *E*. *coli* particles; and interestingly, CD8 stimulation was also able to repolarize IL4-treated M2 cells to an M1 phenotype. Our data suggest that increased CD8 signaling in the post-stroke brain is primarily associated with microglia/macrophages and can independently drive M1 polarization, and that modulation of CD8 signaling could be a potential target to limit secondary post-stroke brain damage.

## Introduction

Cerebral ischemia induces neuronal and glial cell death, resulting in extensive local inflammation of the brain parenchyma and microvasculature characterized by production of pro-inflammatory mediators, rapid activation of resident microglia and infiltration of peripheral leukocytes in the ischemic lesion, such as neutrophils, monocytes/macrophages and different subtypes of T cells [1]. This extensive leukocyte response is arguably detrimental to the ischemic brain contributing substantially to infarct formation causing secondary brain damage, often described as ischemia/reperfusion injury [2, 3].

Activated microglia and infiltrating macrophages represent a major cell population as part of the post-stroke cerebral immune response. These cells have high plasticity that can quickly assume different functional phenotypes in response to specific microenvironmental triggers. Broadly, microglia/macrophages are grouped together as either belonging to the “classically activated” pro-inflammatory M1 phenotype, which amplifies neurodegeneration, or to the “alternatively activated” anti-inflammatory M2 phenotype [4, 5]. M2 cells are generally important in resolution of the inflammatory response, tissue debris scavenging, tissue remodeling, angiogenesis, and have also been shown to be neuroprotective [5]. We have used the traditional M1 and M2 classification in this paper, but where necessary, we have also specified the context in which the macrophages were activated following recent nomenclature guidelines on human and mouse macrophages [6].

Most of the data supports a detrimental role of microglia/macrophages in secondary infarct development [5, 7–10], however, other studies also show their beneficial effect [11, 12]. While the precise cause for this is unknown, different factors could play a role here such as precise post-stroke period analyzed, extent of ischemic injury, the genetic background of mice favoring M1 or M2 responses, and presence of co-morbidity such as infection [4, 13]. More important in this respect is that MCAO mice housed in conventional facilities have been shown to develop spontaneous pneumonia with or without septicemia after 3 days of infarction [13] suggesting that development of post-stroke infection could be an important confounder here and can influence both cell polarization phenotypes as well as disease outcome.

Several studies show that microglia/macrophages are initially polarized towards an M2 phenotype that exerts a beneficial role in prevention of hemorrhagic transformation [9, 14]. However, primed by ischemic neurons, the post-stroke immune responses quickly adapt to a pro-inflammatory state driving the phenotypic polarization of microglia/macrophages towards the M1 phenotype [14]. Moreover, several studies have supported the premise that preserving and reinforcing the initial M2 response could be a promising therapeutic strategy by demonstrating that peripheral administration of M2 macrophages or of M2-polarizing cytokines such as IL-4 generally lead to decreased infarct size and an improved neurological outcome [15, 16]. Alternatively, local M1 polarization could be targeted to modulate the M1/M2 balance towards the M2 phenotype in the post-ischemic brain. The classical M1 activation pathway involves binding of IFNγ to the IFNγ receptor to activate the JAK-STAT1 intracellular signaling pathway [17]. However, other activation pathways involved in macrophage polarization in the brain after stroke are also described that include toll-like receptor (TLR)-4/TNF receptor-associated factor (TRAF)-6 pathway, TLR3/interferon regulatory factor (IRF)-3 pathway, or spleen tyrosine kinase (SYK) signaling through either CD8, FcγR or Clec4 receptors [18–20]. These signaling pathways converge in activation of NF-κB, resulting in increased expression of pro-inflammatory cytokines. NF-κB is also downstream of mammalian target of rapamycin complex 1 (mTORC1), another kinase identified to be involved in M1 polarization in the post-stroke brain [21]. Similarly, CD8-expressing microglia and macrophages have also been observed in the brain after stroke and other CNS injuries [22, 23], however, its significance especially in context with other M1 signaling pathways in the post-stroke brain remains unclear.

In this study, we used a rat MCAO model to investigate the relation of the M2 to M1 microglia/macrophage switch with CD8 expression and the capacity of CD8 to induce the M1 phenotype. We show here that CD8 signaling is an important pathway during M1 polarization in post-stroke rat brain.

## Material and methods

### Animals

The study was conducted according to the guidelines of the Federation of European Laboratory Animal Science Associations (FELASA) and the EU Directive 2010/63/EU for animal experiments. Animal experimentation protocols were approved by the University of Antwerp ethics committee. Studies were performed on mixed gender Wistar rats aged 8–12 weeks with mean weight of 271 ± 39 g. Animals had *ad libitum* access to food and water before and after surgery and were housed in groups until 2 days before surgery, when they were housed individually. Animals that were febrile and/or showed histological evidence of pneumonia, as described by us previously [24, 25], were excluded from the study. Animals belonged to sham or experimental stroke group (n = 6 per group for each study time point) and healthy control group (n = 6). Peritoneal macrophages were isolated from a separate set of healthy control animals (n = 6).

### Middle cerebral artery occlusion

Anesthesia was induced by i.p. injection of ketamine (Anesketin, 55 mg/kg) and medetomidine (Domitor, 0.5 mg/kg). Animals were placed on a heating pad and body temperature and heart rate were monitored (RightTEMP and MouseSTAT, Kent Scientific). Cerebral ischemia was induced for 75 min by middle cerebral artery occlusion using an intraluminal filament as previously described [13, 26]. Decrease of cerebral blood flow was visualized *in vivo* by laser Doppler flowmetry (Periflux, Perimed). In sham-operated animals, the filament was immediately withdrawn by 7 mm to avoid ischemia. The animals were kept under anesthesia during the procedure. All animals were monitored in heated cages for 2 hours after surgery.

### Tissue collection & processing

Animals were euthanized by isoflurane overdose at the fixed study endpoints and tissue was collected. After collection, brains were either processed for histology or snap frozen for transcript analysis. For the former, whole brains were fixed in 4% paraformaldehyde overnight and embedded in paraffin. For the latter, perilesional brain regions were carefully dissected under a stereomicroscope (Stemi SV 11 Zeiss). In addition, left lung was collected and prepared for histology, right lung was homogenized by a tissue homogenizer (Ika, Staufen) in cold sterile PBS, log-diluted and spirally plated on Mueller-Hinton agar plates (Eddy Jet, IUL Instruments) for bacterial enumeration. Plates were incubated at 37°C and colonies counted the next day. Bacterial species were identified by MALDI-TOF mass spectrometry (Bruker) using MALDI Biotyper 3.0 software. Investigators were blinded for the experimental group assignments for all downstream analyses.

### Neurobehavioral scoring

Clinical signs of stroke were assessed by daily neurological evaluation of six parameters: i) spontaneous activity assessment, ii) symmetry in the movement of the four limbs, iii) forepaw outstretching, iv) grip strength, v) body proprioception, and vi) response to vibrissae touch, as described previously [27].

### Histology and immunohistochemistry

For histology, gross infarcts were studied by TTC (2,3,5-triphenyltetrazolium chloride, Sigma-Aldrich) staining on 2 mm coronal fresh brain sections and by H&E staining on 5-μm-thick paraffin sections. Infarct sizes were measured using ImageJ 1.48v on 9 non-sequential sections per brain between bregma +1 and -3 mm and is presented as percentage of the total ipsilateral hemisphere. For immunohistochemical cell analysis, 3 non-sequential sections per brain spaced 100 μm apart were selected from the infarct region between bregma +1 and -3 mm. Immunohistochemistry and immunocytochemistry was performed utilizing the following antibodies: rabbit anti-Iba1 (1:500, 019–19741, Wako), rabbit anti-GFAP (1:15000, Z0334, Dako), mouse anti-CD68 (1:200, MCA341R, Abd Serotec), goat anti-arginase-1 (1:400, Sc-18354, Santa Cruz), rabbit anti-iNOS (1:100, ab15323, Abcam), mouse anti-CD8 (1:1000, MCA48R, Biorad) and rabbit anti-Ki67 (1:1000, ab15580, Abcam), as previously described [28]. Briefly, after pre-incubation with serum for 30 min at room temperature, primary antibodies were incubated overnight at 4°C and visualized with FITC-, cy3-, or cy5-conjugated secondary antibodies incubated for 30 min at room temperature. DAPI (4,6-Diamidino-2-phenylindole, Sigma-Aldrich) was used as a nuclear counterstain. Immunofluorescent double labeling of same-host antibodies was performed using a diluted first primary antibody (CD8 1:5000 final dilution) detected with a 5 min tyramide-Alexa Fluor 488 (Thermo Fisher) amplification followed by an antibody elution step, and a direct non-amplified detection of a more concentrated second primary target, as described previously by us [29]. This second detection method did not cross react with the first primary antibody (Supplementary information, S1 Fig). Images were taken on a dual spinning disk confocal microscope (Ultra*View* VoX, PerkinElmer) at 200 x magnification and 8 images per section, 3 sections per brain were analyzed using Volocity (Perkin Elmer). Data are expressed as proportions and/or as mean numbers of cells per high power field (0.16mm^2^). Single labeling for CD68, Iba1 and GFAP was visualized by DAB (5’, 5’ diaminobenzidine, Dako)-stained sections with hematoxylin counterstaining and images were acquired on an Axioscope AX10 light microscope (Zeiss) equipped with a CCD UC30 camera (Olympus)[28].

### Transcript analysis

RNA extraction of frozen regions of interest was performed using the RNAeasy Mini kit (Qiagen) after homogenization in liquid nitrogen. RNA integrity and concentrations were estimated using RNA nanochips on Bio-analyzer (Agilent) and converted to cDNA using the RT^2^ First Strand kit (Qiagen). Quantitative PCR was performed using Sso Advanced SYBR green supermix (Biorad) using 2-step PCR with cycles of 95°C for 10 sec followed by 60°C for 30 sec. Following transcripts were analyzed: *Il-6*, *Tnfα*, *Il-1α*, *Il-1β*, *Il-18*, *Rorc*, *Il-17a*, *Il-17f*, *Icam*, *Mac-1*, *Cd86*, *Cd8*, *FcγR*, *Clec4*, *IfnγR*, *Tlr3*, *Tlr4*, *Syk*, *Stat1*, *Irf3*, *Traf6*, *Raptor*, *Actb*, *Sdha* using custom and commercial PCR-arrays (Qiagen) based on initial analysis (primer sequences available upon request). Data were analyzed using the comparative C_T_ method and reported as fold changes vs. sham control, as described earlier [30, 31].

### Macrophage culture, cell stimulation, and phagocytosis assay

Peripheral macrophages were isolated by lavage from the peritoneal cavity with ice-cold Mg^2+^ and Ca2+—free PBS. Cells were centrifuged at 450 x g for 10 min and resuspended in DMEM-F12/10. Cells were seeded at density of 2 x 10^4^ per well of a 24-well tissue culture plate at 37°C with 5% CO_2_. Cells were further stimulated for 24 h by either CD8 (1 μg/well in 100 μl of culture medium, OX8, eBioscience) or IgG1 isotype control (1 μg/well, eBioscience). Additional subsets of cells were further stimulated for 6 h using LPS (20 ng/mL, Sigma-Aldrich). Phagocytosis assay was performed using labeled *E*. *coli* particles, according to the manufacturer’s specifications (Cytoselect, Cell Biolabs). After medium refreshment, measured amounts of *E*. *coli* particles were added to the wells and incubated for 6 h at 37°C with 5% CO_2_ followed by colorimetric detection at 450 nm. In separate experiments, cells were induce the M2 phenotype [32] by pre-treated for 48 h with recombinant IL-4 (2 ng in 100 μL culture medium per well, Thermo-Fischer), which was followed by CD8 stimulation with phagocytosis assay performed as described above.

### Data analysis and statistics

Data analyses were performed using SPSS version 21 (IBM, USA). Transcript data are presented as average fold differences with standard errors of the mean and immunohistochemical and phagocytic data are presented as averages normalized to control with standard errors of the mean. Differences between groups were tested using a 2-tailed independent *t*-test, and data were log transformed for transcript data. When comparing multiple time points, statistical significance was tested by one-way ANOVA with post hoc Bonferroni correction. *P* < 0.05 was considered statistically significant. For PCR array data, post hoc Bonferroni correction was applied.

## Results

### M1 polarized microglia/macrophages predominate in the perilesional areas 4 days after ischemia/reperfusion

**Fig 1:**
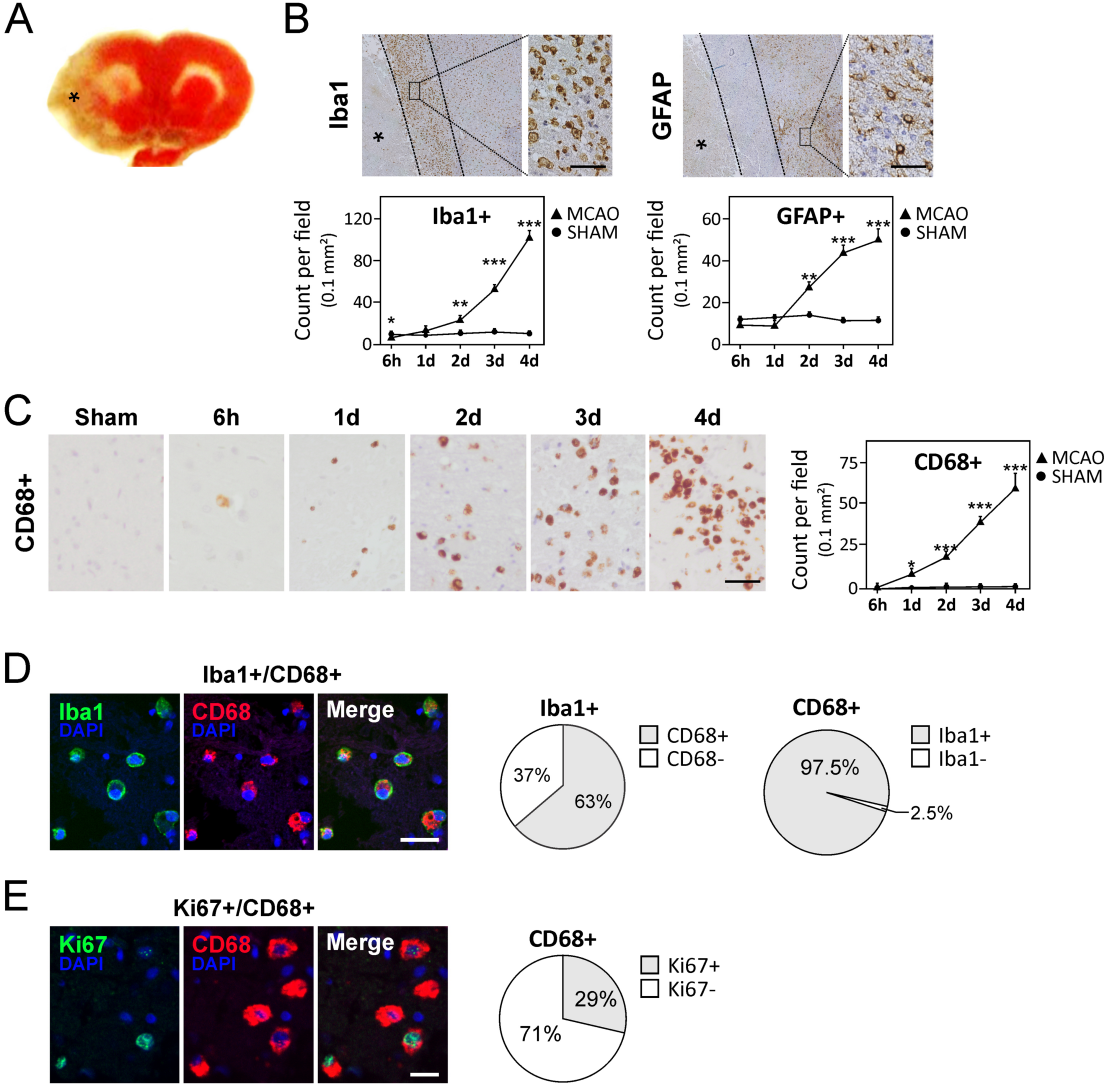
Activated microglia/macrophages accumulate in the perilesional areas after MCAO. **(A)** TTC-stained brain section indicating the stroke area (pale color marked by asterisk). **(B)** Upper panels show glial scar at day 4 in the perilesional area stained for microglia (Iba1) and astroglia (GFAP) that do not strictly overlap. Lower panels show quantifications of Iba1 and GFAP reactive cells in the glial scar region for different time points. Asterisks mark the infarct area. (*See* S2 Fig *for different coronal slices of stroke brain*). **(C)** Progressive accumulation of activated microglia/macrophages in the glial scar region over 4 days. **(D)** Representative images of Iba1/CD68 co-staining and quantification at day 4 after ischemic injury. **(E)** Representative images of Ki67/ CD68 immunofluorescent co-staining and quantification at day 4 after ischemic injury. Scale bars in *B* and *C* represent 32 μm and data are presented as average counts ± SEM per field of 0.1 mm^2^. Scale bars in *D* and *E* represent 18 μm and field sizes of 0.125 mm^2^. * *P* < 0.05; ** *P* < 0.01; *** *P* < 0.001.

To study microglia/macrophage activation and polarization after ischemic stroke, we utilized the well-established MCAO rat model [13, 26], which showed large ischemic lesions in the ipsilateral cortex and striatum (Fig 1A) and corroborated with the strong neurobehavioral decline observed in these animals (S2A and S2B Fig). A total of 10 animals developing spontaneous pneumonia were excluded and in lungs of these animals, endogenous commensal gut bacteria were identified by MALDI-TOF as main etiologic agent. Brain transcript studies showed an acute upregulation of pro-inflammatory cytokines (*Il-6*, *Tnfα*, *Il-1α*, *Il-1β*, and *Il-18*; S3A Fig), as shown previously [33]. At day 4 after injury induction, expression of *Tnfα*, *Il-6*, and *Il-18* remained elevated. Also at this time point, brain transcript levels of *Il-17* and it transcriptional factor, *Rorc*, became significantly upregulated (S3A Fig). Moreover, *Icam1*, a marker for leukocyte recruitment, was actively induced after infarction (S3B Fig). Immunohistochemical staining of GFAP and Iba1 on consecutive sections showed development of the glial scar at the perilesional areas of the infarct (Fig 1B).

To study the impact of activated microglia/macrophages in development of the glial scar, we performed double labeling of Iba1 with macrophage activation marker, CD68, and showed that 63% of Iba1+ cells were activated microglia/macrophages at the perilesional areas (Fig 1D). By quantitative immunohistochemistry, we further showed a gradual increase in activated CD68+ cells between 6 hours and 4 days following stroke (Fig 1C) that corroborated with increased transcripts of a general macrophage marker, *Mac-1* (*P* < 0.001; S3B Fig). As brain phagocytes are known to proliferate in the subacute phase of stroke [34, 35], we co-stained for Iba1 and proliferation marker Ki67 for day 4 and identified 42% cells to be Ki67+Iba1+ (S4 Fig). Similar experiments for activated macrophage marker CD68 showed 29% Ki67+CD68+ cells for day 4 (Fig 1E). These data indicate that CD8 expression is preserved on proliferating brain phagocytes.

**Fig 2:**
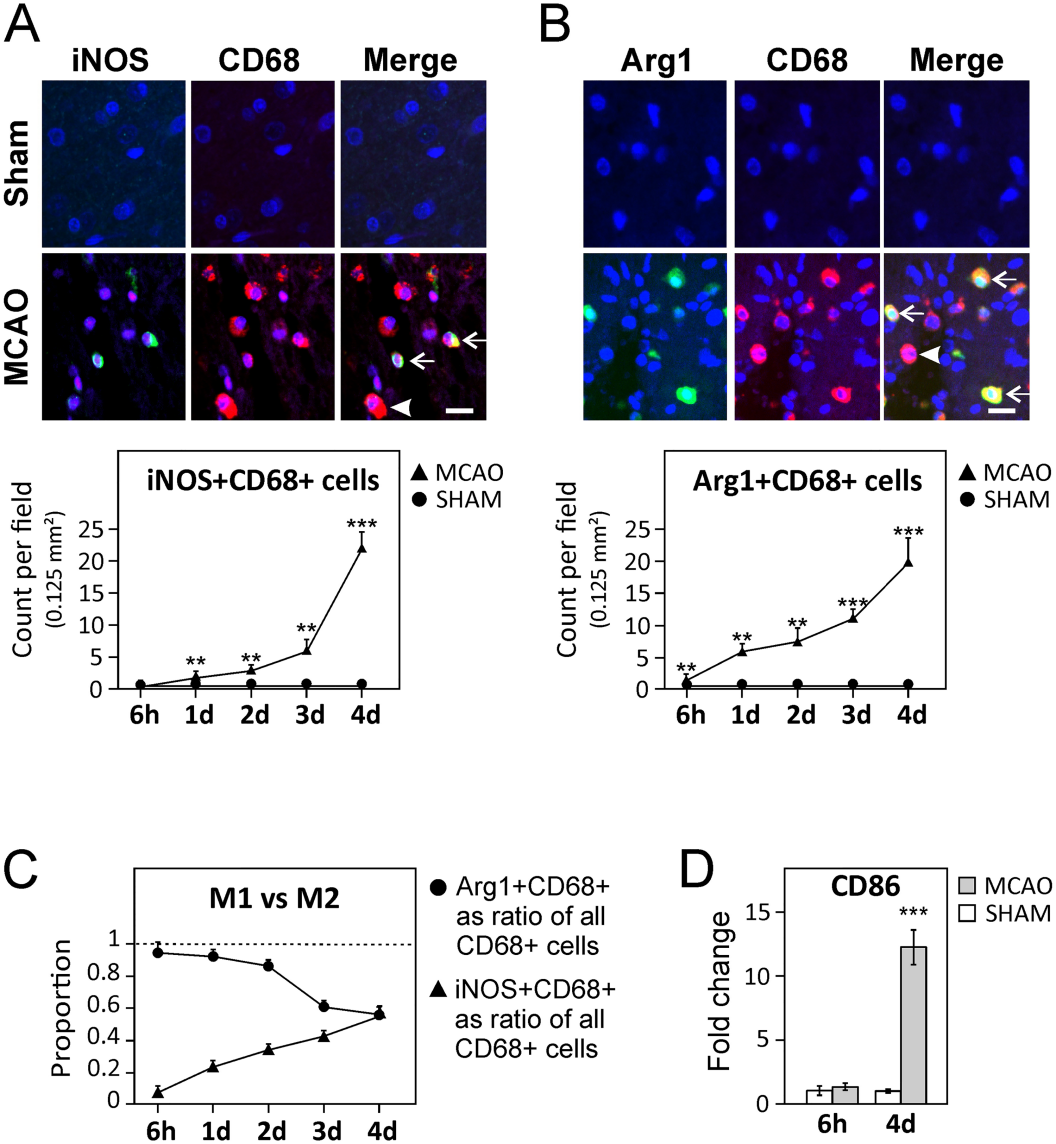
M2 microglia/macrophages are recruited early after MCAO and progress towards M1 phenotype. (**A–B**) Upper panels are representative double-labeled immunohistochemistry for iNOS+CD68+ (M1, ***A***) and Arg1+CD68+ (M2, ***B***) cells in the infarct border after stroke. Arrows indicate double positive cells and arrowheads indicate CD68+ cells not stained with Arg1 or iNOS, respectively. Scale bars represent 16 μm. The quantification of these cells at different time points are presented in the lower panels. Data are presented as average (Av) counts ± SEM per field of 0.125 mm^2^ (**C**) iNOS+CD68+ and Arg1+CD68+ cells shown as a proportion of total CD68+ cell population. (**D**) The dramatic increase in M1 cells by 4 days after stroke was confirmed by brain transcript analysis of M1 marker Cd86/B7-2. *A*–*D*: Data are presented as mean ± SEM. * *P* < 0.05; ** *P* < 0.01; *** *P* < 0.001.

To study the evolution of polarization of activated microglia/macrophages after MCAO, representative M1-associated (inducible nitric oxide synthase, iNOS) and M2-associated (arginase-1, Arg1) protein markers were analyzed along with an activated microglia/macrophage marker (CD68) by immunofluorescent staining. Recruitment of Arg1+CD68+ (M2) cells to the perilesional area was observed within 1 day after MCAO that increased until day 4 (Fig 2B). In contrast, the increase in iNOS+CD68+ cells showed a delayed profile with a very modest increase before 48 h after stroke, but increasing substantially between days 2 and 4 after stroke (Fig 2A). Looking at the proportional contribution of the iNOS+ and Arg1+ subsets to the total number of activated CD68+ microglia/macrophages, more than 90% of CD68+ cells were Arg1+ in the early stages after stroke; however, these numbers decreased substantially by day 4, where they constitute ≈55% of all activated microglia/macrophages (Fig 2C). In contrast, the proportion of iNOS+CD68+ increased from less than 10% at 6 h time point to 55% at day 4 (Fig 2C). Additionally, a small proportion of CD68+ cells stained positive for both Arg1+ and iNOS+ (S5 Fig). The observed increase in activated M1 cells at day 4 post-stroke was further confirmed by transcript analysis of perilesional areas of the M1 marker Cd86/B7-2 (*P* < 0.001) (Fig 2D). These data suggest that while both M1 and M2 subsets are being actively recruited to the ischemic area, M1 cells proportional increase whereas M2 cells proportionally decrease after stroke.

### CD8-mediated signaling is involved in M1 polarization after stroke

**Fig 3:**
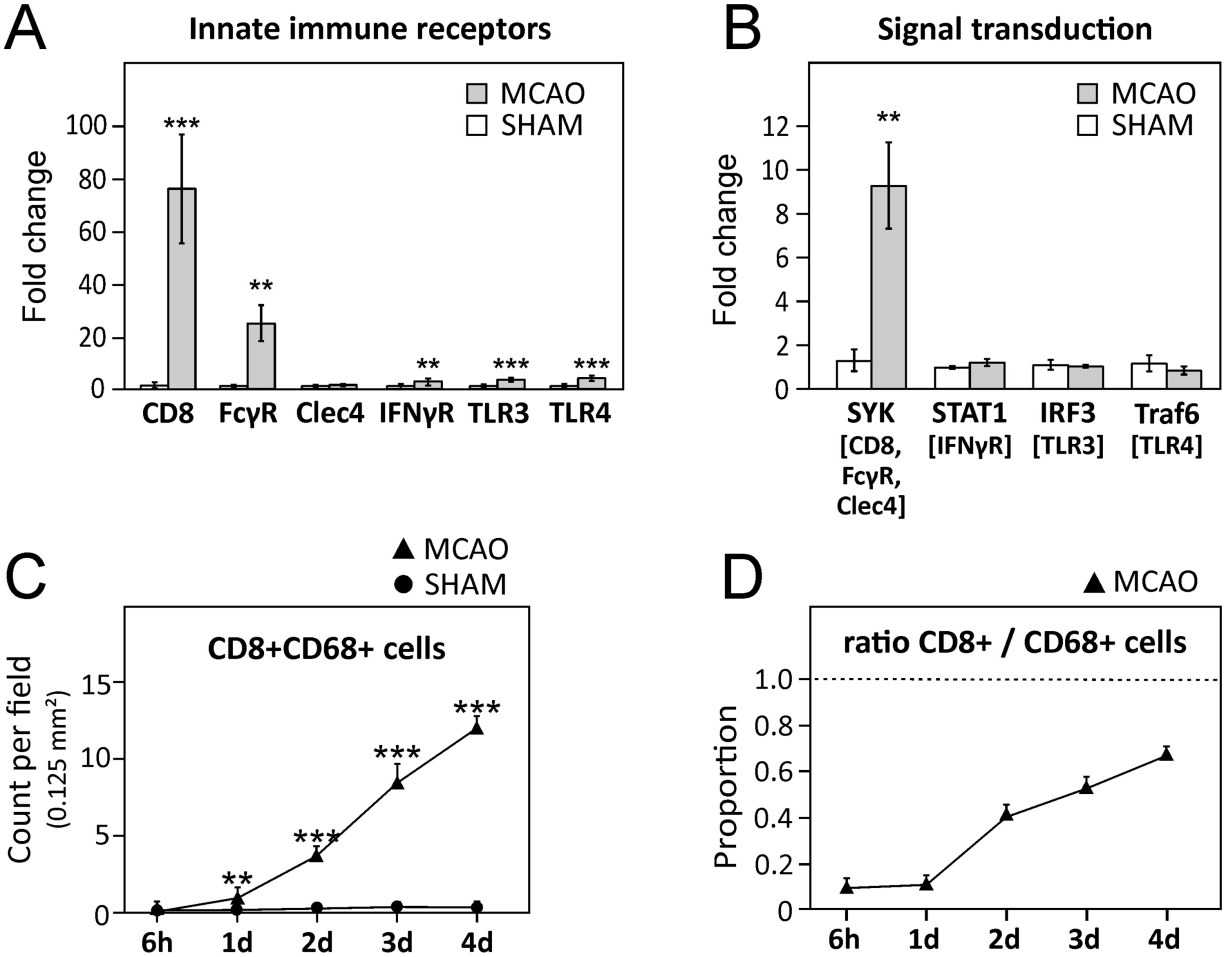
CD8 signaling in post-stroke brain. **(A)** Expression of innate immune receptors and of **(B)** signal transduction molecules of respective pathways (indicated in brackets) involved in M1 polarization analyzed 4 days after stroke in the perilesional areas of the ischemic hemisphere. **(C)** Quantification of CD8+CD68+ cells as average (Av) counts ± SEM per field of 0.125 mm^2^ **(D)** Proportion of CD68+ cells expressing CD8 for MCAO animals. Sham animals are not plotted that lacked either or both CD8+ or CD68+ cells showed ratio was either 0 or a/0. *A*-*D*: Data are presented as mean ± SEM. * *P* < 0.05; ** *P* < 0.01; *** *P* < 0.001.

The M1 phenotype is induced by different pathways activated by different receptors, namely LPS-R/TLR4, TLR3, IFNγR, FcγR, Clec4, and CD8 [18–20]. Using transcript analysis, we first studied the expression of these signal receptors in perilesional dissected brain regions at day 4 after stroke. While *Clec4* was not elevated and *Tlr4*, *Tlr3* and *IfnγR* showed a moderate but significantly increased expression in the injured hemisphere (5-fold, *P* < 0.001; 4-fold, *P* < 0.001; 4-fold, *P* < 0.01, respectively), *FcRγ* was elevated by ≈20 fold (*P* < 0.01). However, the increase in CD8 was most dramatic, increasing by 77-fold in post stroke brain (*P* < 0.001; Fig 3A). We further studied the expression of key signal transduction molecules of the different studied M1 stimulatory pathways (*Syk*, *Stat1*, *Irf3*, and *Traf6*), and showed a significant 9-fold increased expression of *Syk*, a central molecule in CD8, FcγR, and Clec4 signal transduction pathway (*P* < 0.001; Fig 3B). These data suggest that CD8 signaling is an important signaling mechanism in post-stroke brain.

We further studied the cell types that expressed CD8 in post-stroke brain. Using double immunofluorescent labeling, we showed that CD8+CD68+ cells rapidly increased after stroke from almost being non-detectable at 6 h after stroke (Fig 3C) to be expressed on 67% of all CD68+ cells at day 4 (Fig 3D). We also identified Ki67 reactivity in 30% of CD8+Iba1+ cells suggesting that a proportion of CD8+Iba1+ are proliferating (S4 Fig). Considering that CD8 is primarily a T-cell marker, we also CD8 staining with a pan-T-lymphocyte cell marker, CD3 (S5 Fig). In accordance with earlier observations [23, 36], large infiltration of CD8+ cells was observed while CD3-positive T-lymphocytes were sporadically observed. These data suggest that CD8 reactivity is predominantly present on CD68+ microglia/macrophages in post-stroke brain.

**Fig 4:**
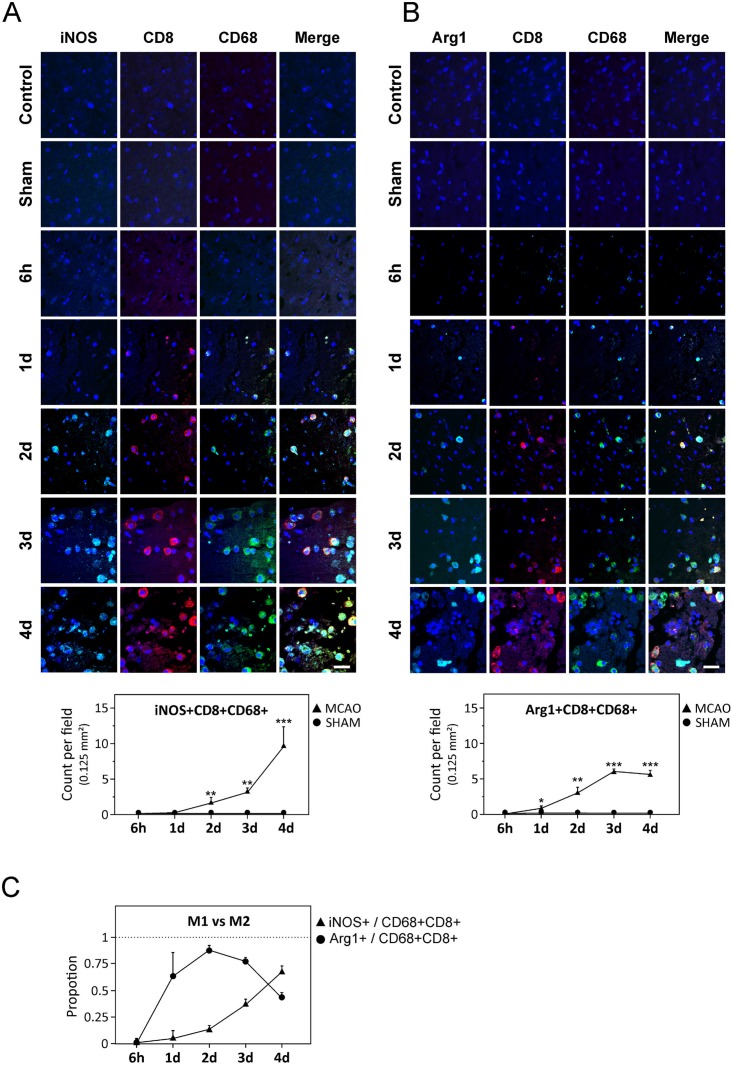
CD8-expressing cells in post-stroke brain. **(A–B)** Upper panel shows representative triple-labeled immunostaining of CD8+CD68+ cells expressing iNOS or Arg1 in the perilesional areas of the ischemic hemisphere at 6 h, and at days 1, 2, 3 and 4 after stroke. Lower panel shows quantification data. **(C)** Proportion of CD8+CD68+ cells expressing either Arg1 or iNOS at 4 d after stroke. Data in *A*–*C* is presented as average ± SEM. Scale bars represent 32 μm. * *P* < 0.05; ** *P* < 0.01; *** *P* < 0.001.

Following the premise that CD8 is involved in M1 polarization in stroke, we further studied the evolution of the M1/M2 markers on CD8+CD68+ cells. The number of iNOS+CD8+CD68+ cells strongly increased from day 2 onwards and at day 4, 67% of all CD8+CD68+ cells were iNOS+ (Fig 4A). On the other hand, Arg1+CD8+CD68+ cells increased until day 3 but declined thereafter (Fig 4B) and proportion wise, iNOS+CD8+CD68+ cells became the most dominant CD8+CD68+ cell type at day 4 after stroke (Fig 4C). These data suggest a strong correlation between CD8 signaling and M1 polarization after stroke.

### CD8 stimulation primes macrophages towards M1-type functionality

**Fig 5:**
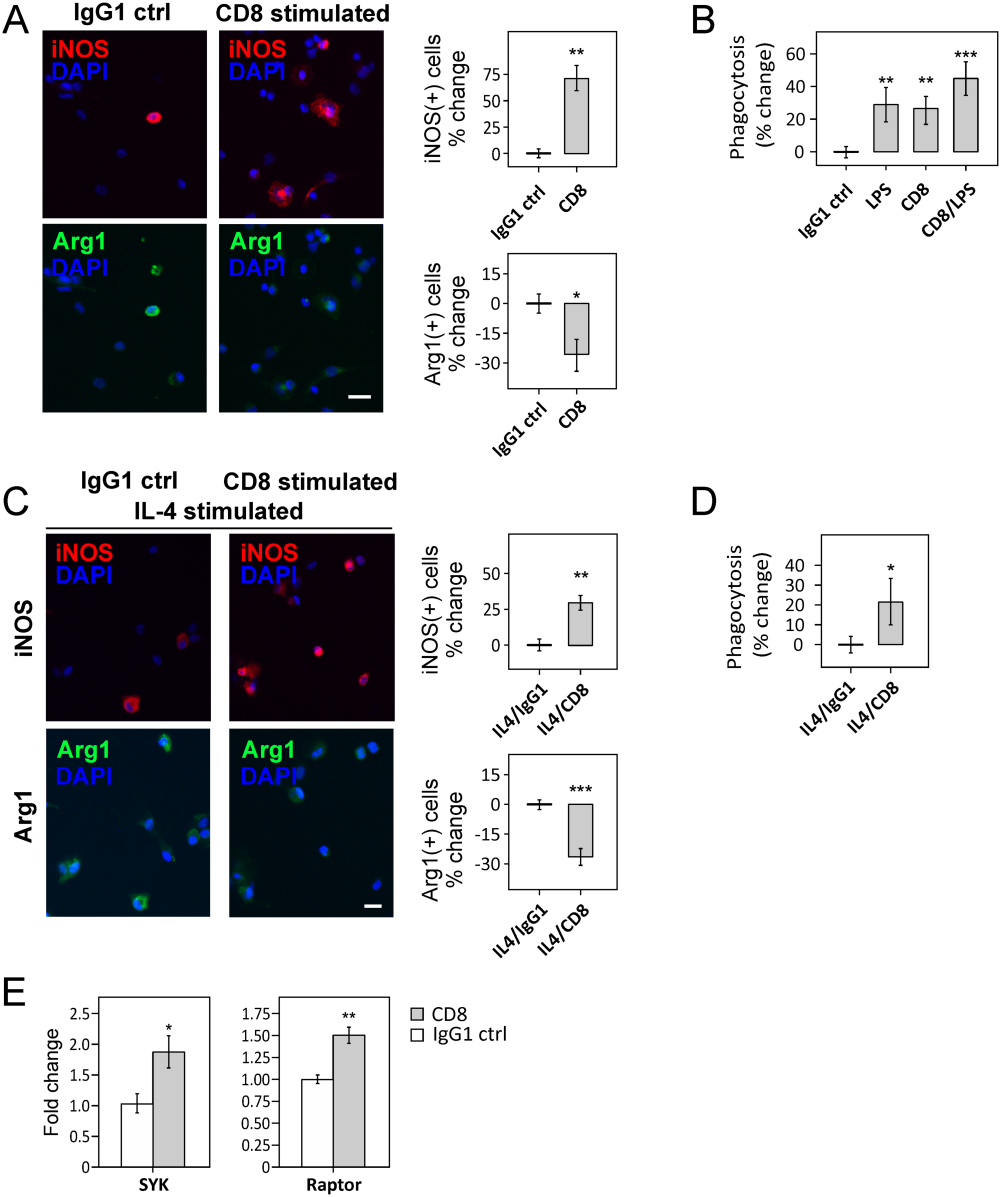
CD8 stimulation induces M1 phenotype. (**A**) Left panel shows increased iNOS+ and decreased Arg1+ staining in cultured peripheral macrophages after 24h with CD8-stimulating ligand, compared to the IgG1 isotype control. Right panel shows quantification of iNOS+ and Arg1+ cells presented as percentage increase or decrease respectively over the IgG1 control condition. (**B**) Increased phagocytosis of *E*. *coli* particles after CD8 or CD8-LPS stimulation by macrophages in *in vitro* assays. (**C**) IL-4-stimulated cells, when further stimulated with a CD8-stimulating ligand, showed increased iNOS and decreased Arg1 expression (left panel). Right panel shows quantification of iNOS+ and Arg1+ cells presented as percentage increase or decrease respectively over the IgG1 control condition. (**D**) Increased phagocytosis of *E*. *coli* particles with IL-4-CD8-stimulated macrophages compared to the IL-4-IgG1 isotype control. (**E**) Increased expression of signal molecules *Syk* and *Raptor* after CD8 stimulation. Data presented in *A*–*E* are normalized to control and presented as average ± SEM. * *P* < 0.05; ** *P* < 0.01; *** *P* < 0.001. A, C, D: Scale bars represent 18 μm.

To investigate whether CD8 presence on post-stroke macrophages is directly involved in M1 phenotypic polarization, rat peripheral macrophages were isolated and treated with a CD8-stimulating ligand [18]. After 24 h stimulation, iNOS expression on CD8-stimulated cells M(CD8) increased by 72% (*P* < 0.01), compared to isotype (IgG1)-stimulated controls while Arg1 expression decreased by 27% (*P* < 0.05, Fig 5A). To further investigate whether CD8 stimulation has a direct effect on macrophage function, we examined *in vitro* phagocytic capabilities using labeled *E*. *coli* particles. CD8+ cells showed a 26% increase in phagocytosis (*P* < 0.01) and was comparable to phagocytic capacity of LPS stimulated macrophages (29%; Fig 5B), the latter commonly employed for studies of M1 activation [37].

We further investigated whether CD8 stimulation was also able to repolarize IL-4-induced M2 macrophages towards the M1 phenotype. As expected, macrophages induced with IL-4 for 2 days (M(IL-4)) showed a 61% increase in Arg1 expression (*P* < 0.01) compared to the non-stimulated control condition (S7A Fig), while iNOS expression was unchanged. After induction with IL-4, macrophages showed a decreased capability for phagocytosis of *E*. *coli* particles (S7B Fig). Further stimulating M(IL-4) with CD8 resulted in a 30% increased iNOS expression, 28% decreased Arg1 expression (Fig 5C) and 22% increased phagocytic activity, compared to M(IL-4) cells treated with IgG1 isotype control (*P* < 0.05; Fig 5D). Interestingly, LPS-stimulated macrophages (M(LPS)) when co-stimulated with CD8 showed an increase in phagocytic activity by 20% compared to the IgG1 control situation (*P* < 0.001), suggesting a synergistic effect of CD8 and LPS stimulation (Fig 5B). We also show that specific stimulation of CD8 alone resulted in upregulation of its signal transducers *Syk* (P < 0.05) and *Raptor* (*P* < 0.01; Fig 5E), the latter being part of the mTORC1 complex shown to be involved in stroke-related inflammation [21]. These data suggest that CD8 stimulation can independently drive an M1 phenotype and has the potential to independently repolarize M2 cells towards an M1 phenotype.

## Discussion

Microglia and infiltrating peripheral macrophages are among the first responders to cerebral ischemic injury and are important mediators of the immune response, playing an important role in both expansion of neuronal injury and tissue recovery in post-ischemic brain [12, 38]. In this respect, general depletion of microglia and brain macrophages has been linked to both increased hemorrhagic conversion as well as infarct reduction in experimental models of stroke [11]. Moreover, peripheral or central administration of M1 and M2 cells have also been shown to have beneficial, deleterious or no effect, suggesting that the evolution and functions of different subsets of microglia and brain macrophages in post-stroke brain are more complex than initially thought [15, 39, 40]. In this study, ≈50% of animals by day 3–4 developed pneumonia [24, 25]. These data suggest that spontaneous development of infection due to stroke-induced immunosuppression, also shown earlier [13], is a major confounding factor for macrophage polarization studies.

Excluding animals developing pneumonia, we showed that the majority of activated microglia/macrophages recruited to the injured brain area immediately after the ischemic infarct have an M2 phenotype. We did not attempt to make a distinction between microglia and macrophages in this study, as both microglia [34, 35] and infiltrating macrophages [23, 41] have been shown to contribute heavily to this population. For instance, while one recent study utilizing a parabiotic CX3CR1^GFP/-^ model showed that microglia accounted for the majority of mononuclear phagocytes after stroke [35], another recent study utilizing BRDU incorporation into bone marrow monocytes showed that after 72 hours, microglia did not proliferate and that the majority of mononuclear phagocytes were peripheral macrophages [41]. While the precise reason for this is unknown, various factors including the stage (acute versus subacute) or extent of acute ischemic injury might play a role here. We showed that not only 42% of Iba1+ cells were proliferating at day 4, a proportion of Iba1+CD8+ cells were also expressing Ki67, suggesting that CD8+ is preserved on proliferating brain phagocytes. Because we did not use a discriminating marker between microglia and macrophages, we cannot be certain that Ki67+ brain phagocytes are indeed microglia, however, infiltrating phagocytes in brain are not observed to proliferate [35].

While the relative contribution of microglia and macrophages to the monocyte derived phagocytes in the brain after stroke remains to be fully elucidated, both pro-inflammatory M1 microglia and macrophages have also been observed in post-stroke brain, where they are shown to contribute to secondary expansion of the ischemic injury [14, 42, 43]. Nevertheless, several studies applying therapeutic approaches to specifically strengthen the protective M2 responses, by introducing M2-polarized cells or M2-polarizing cytokines such as IL-4, have shown mixed results. For instance, stimulating the M2 response in the acute (<12h) [16] or the recovery (>7 days) phase [15] of stroke has been shown to improve neurological outcome. However, administration of M2 cells in the subacute phase (4 days after injury onset) showed no difference in infarct size and neurological outcome in rodents [39]. While the precise reasons for this are unknown, we show here that M1 polarized cells quickly become a co-dominant phenotype by day 4 after injury onset. Thus, one of the possibilities is that the strong pro-inflammatory milieu at this time point coerces a rapid polarization of therapeutically delivered M2 cells to M1 cells, rendering the cellular intervention ineffective. A brain M2 to M1 polarization due to local factors rather than infiltration of M1 cells in the post-stroke brain is also supported by the fact that stroke induces peripheral immunosuppression, characterized by a switch from Th1 to Th2 cells [13]. In this context, ischemic neurons have recently been suggested as one of the factors that prime microglia/macrophage polarization towards an M1 phenotype [14]. While this paper was under review, a study by Schmidt and colleagues showed that macrophage transfer of M1 or M2 has no impact on outcome after ischemic stroke in mice, also suggesting that local transformation of the M2 macrophages towards the M1 phenotype could be a cause [40].

Different cell-surface receptors and cell-signalling cascades are identified in M1 polarization, however, their specific role in the post-stroke brain are debated [18–20, 44]. In the current study, we examined several receptors and their downstream key signaling molecules involved in M1 polarization and identified an important role for CD8 signaling in post-stroke brain. While CD8 is mainly known as a T-cell marker, presence of CD8 receptor on the cell surface of alveolar macrophages has also been observed in both rats and humans [18, 45] where they cause increased production of pro-inflammatory markers [18, 45, 46]. CD8 expressing microglia/macrophages have also been observed in various CNS disorders, including stroke, however, the significance of these findings remained unclear [22, 23]. We showed here ≈80-fold increase of CD8 brain transcript levels in microglia/macrophage-rich post-stroke perilesional brain areas that corroborated the immunohistochemical data of strong CD8 receptor expression on microglia/macrophages. Moreover, despite the fact that alterations in the transcript levels of nuclear transcription factors can be diluted in tissue analysis, we showed here a significant upregulation of the CD8 signal transducer *Syk*, in contrast to transcriptional factors involved in other pathways. While the signal transducer *Syk* is also downstream of other signal receptors in the ischemic brain such as FcγR and Clec4, we did not observe upregulation of Clec4, and although FcγR was significantly increased, the increase in FcγR was still 4-fold lower than increase in CD8 expression. These data strongly suggest that CD8 signaling is robustly activated in post-stroke rat brain.

We also showed that CD8 receptor expression was strongly associated with the M1 phenotype in the perilesional areas of the post-stroke brain whereby an increase in CD8 transcripts and immunohistochemical staining strongly correlated with decreasing Arg+CD8+CD68+ cells and increasing iNOS+CD8+CD68+ cells. This association also had a causal relationship as naïve macrophages, when stimulated *in vitro* with CD8-stimulating ligand, upregulated the downstream transducer *Syk*, increased their iNOS expression, and phagocytosed bacterial particles more efficiently. We also showed that stimulation of CD8 signaling in IL-4 treated M2 macrophages [14, 32] could also repolarize them to an M1 phenotype with enhanced phagocytic potential towards bacterial particles. These data suggest that the increased expression of CD8 on microglia/macrophages in stroke brain has a functional relevance as CD8 signaling can independently polarize microglia and macrophages towards a functional M1 phenotype.

Lastly, we showed in CD8-stimulated macrophages, increased transcript levels of Raptor, a key component in mTORC1 signaling. As a central modulator, Raptor/mTORC1 is activated by Syk but can also interact with other signaling molecules including Traf6 and Stat3 in various cell types, including macrophages [47]. Interestingly, mTORC1 modulates NF-κB pathway [48], and both mTORC1 and NF-κB signaling have been described in post-stroke M1 macrophage polarization [49]. Moreover, mTOR blocking *in vitro* was shown to prevent LPS/TLR4 induced M1 polarization [50]. In our study, we did not observe a major involvement of TLR-4 and its downstream *Traf6* signaling suggesting that CD8 could be a major upstream activator of mTOR/NF-κB in stroke.

To conclude, we show here that CD8 signaling could be an important mechanism for M1 polarization associated with secondary brain damage in the post-stroke brain, and targeting CD8 signaling could be a novel strategy to limit M1-mediated secondary post-stroke injuries.

## Supporting information

S1 Fig Validation of CD8/CD68 double immunofluorescence staining using tyramide-Alexa Fluor 488. Negative control was obtained after treating tissue stained with 5x diluted primary CD8 antibody (1:5000) with secondary DAM-Cy5 showing no visible interaction between the fluorescent secondary antibody and the diluted primary otherwise visualized with tyramide-Alexa Fluor 488. Applying secondary DAM-Cy5 at the normal optimized dilution for CD8 (1:1000) shows a visible interaction between the primary and secondary antibody. Scale bar represents 18 μm.
(TIF)

S2 Fig MCAO results in large ischemic lesions in the cortex and striatum and in a neurobehavioral decline. (A) TTC staining showing large infarct areas (pale) induced by MCAO. (B) Experimental stroke caused a decline in neurobehavioral scores, represented by the average score of each group at each time point, (*see* Methods). Data are presented as average ± SEM. * *P* < 0.05; ** *P* < 0.01; *** *P* < 0.001. Presurg, baseline score of animals prior to surgery.
(TIF)

S3 Fig MCAO-induced increased expression of pro-inflammatory cytokines in the ischemic brain region. (A) Transcript analysis of ischemic brain region shows strong upregulation of pro-inflammatory cytokines *IL-1α*, *IL-1β* at 6 h after MCAO that declines by day 4, while expression of *TNF-α*, *IL-6* and *IL-18* was consistently upregulated. *RORC* and *IL-17A/F* were both upregulated at day 4 after injury. (B) Transcript analysis of ischemic brain region at 6 h and 4 days after MCAO showed increased expression of Mac-1 and ICAM at day 4. *A*, *B* Data are presented as fold differences relative to sham-operated animals of the same time point as average ± SE. * *P* < 0.05; ** *P* < 0.01; *** *P* < 0.001.
(TIF)

S4 Fig Proliferating Ki67+ microglia in the stroke brain. Immunofluorescent co-staining of Ki67, Iba1 and CD8 at day 4 after ischemic injury. Arrowheads indicate Ki67+Iba1+ cells and arrows indicate Ki67+CD8+Iba1+ triple positive cells. Scale bar represents 18 μm and field of 0.125 mm^2^.
(TIF)

S5 Fig Arginase-1 and iNOS co-positive CD68+ cells in stroke brain. Left panel shows representative immunofluorescent staining of iNOS and Arg1 expression in CD68+ cells in the perilesional areas at day 4 after cerebral ischemic injury. Arrowheads indicate CD68+ cells positive for both iNOS and Arg1. Quantification (right panel) showing proportion of Arg1+, iNOS+ and double positive CD68+ cells. Scale bar represents 9 μm and data is presented as average percentage cell count ± SEM.
(TIF)

S6 Fig CD8 and CD3 immunohistochemistry in ischemic brain. Representative staining of CD3+ and CD8+ cells in the striatum in parallel sections of sham and MCAO model 4 days post surgery, illustrating that CD8 expression in the ischemic brain cannot be explained by presence of CD3+ lymphocytes. Scale bars represent 25 μm.
(TIF)

S7 Fig IL-4 stimulation of peripheral macrophages. (A) Representative images from peripheral macrophages isolated from peritoneum and stimulated with IL-4 or vehicle control for 48 h. Cells were stained with iNOS, Arg1 and CD68. IL-4 induced an M2-like phenotype characterized by increased expression of Arg1 while iNOS positive cells were rare and no significant difference between IL-4 stimulation and control was observed. Scale bars represent 36 μm. (B) Quantification of M1 (iNOS+CD68+) and M2 (Arg1+CD68+) macrophages of IL-4 or vehicle control stimulated cultures. (C) Decreased phagocytosis of *E*. *coli* particles after IL-4 stimulation of macrophages compared to vehicle control. *B*, *C* Data presented are normalized to control and presented as average ± SEM. * *P* < 0.05; ** *P* < 0.01; *** *P* < 0.001.
(TIF)

S1 File All data. All datapoints represented in the figures of this manuscript.
(XLSX)
